# Discovery of peptide ligands targeting a specific ubiquitin-like domain–binding site in the deubiquitinase USP11

**DOI:** 10.1074/jbc.RA118.004469

**Published:** 2018-10-29

**Authors:** Anastasios Spiliotopoulos, Lia Blokpoel Ferreras, Ruth M. Densham, Simon G. Caulton, Ben C. Maddison, Joanna R. Morris, James E. Dixon, Kevin C. Gough, Ingrid Dreveny

**Affiliations:** From the ‡ Centre for Biomolecular Sciences, School of Pharmacy, University of Nottingham, Nottingham NG7 2RD,; the § School of Veterinary Medicine and Science, Sutton Bonington Campus, College Road, Sutton Bonington, Leicestershire LE12 5RD,; the ¶ Birmingham Centre for Genome Biology and Institute of Cancer and Genomic Sciences, Medical and Dental Schools, University of Birmingham, Birmingham B15 2TT, and; ‖ ADAS, School of Veterinary Medicine and Science, Bonington Campus, College Road, Sutton Bonington, Leicestershire LE12 5RD, United Kingdom

**Keywords:** peptide interaction, peptides, protease, crystal structure, ubiquitin, ubiquitin-dependent protease, phage display, DNA damage response, deubiquitylation (deubiquitination), cell-penetrating peptide (CPP), molecular recognition, USP11

## Abstract

Ubiquitin-specific proteases (USPs) reverse ubiquitination and regulate virtually all cellular processes. Defined noncatalytic domains in USP4 and USP15 are known to interact with E3 ligases and substrate recruitment factors. No such interactions have been reported for these domains in the paralog USP11, a key regulator of DNA double-strand break repair by homologous recombination. We hypothesized that USP11 domains adjacent to its protease domain harbor unique peptide-binding sites. Here, using a next-generation phage display (NGPD) strategy, combining phage display library screening with next-generation sequencing, we discovered unique USP11-interacting peptide motifs. Isothermal titration calorimetry disclosed that the highest affinity peptides (*K_D_* of ∼10 μm) exhibit exclusive selectivity for USP11 over USP4 and USP15 *in vitro*. Furthermore, a crystal structure of a USP11–peptide complex revealed a previously unknown binding site in USP11's noncatalytic ubiquitin-like (UBL) region. This site interacted with a helical motif and is absent in USP4 and USP15. Reporter assays using USP11-WT *versus* a binding pocket–deficient double mutant disclosed that this binding site modulates USP11's function in homologous recombination–mediated DNA repair. The highest affinity USP11 peptide binder fused to a cellular delivery sequence induced significant nuclear localization and cell cycle arrest in S phase, affecting the viability of different mammalian cell lines. The USP11 peptide ligands and the paralog-specific functional site in USP11 identified here provide a framework for the development of new biochemical tools and therapeutic agents. We propose that an NGPD-based strategy for identifying interacting peptides may be applied also to other cellular targets.

## Introduction

There are ∼54 ubiquitin-specific proteases (USPs)^6^ encoded in the human genome, most of which harbor a canonical cysteine protease catalytic triad. USPs specifically reverse ubiquitination of a range of substrates to regulate virtually all cellular processes, including protein degradation, signaling pathways, DNA damage repair, transcription, and receptor endocytosis (1). USPs harbor a catalytic domain that is flanked or interspersed by additional domains for versatile functionality and specificity (2, 3). For most USPs, interactions involving these domains are poorly understood, and only few selective agents to probe a particular USP's function are available (4–10). Human ubiquitin-specific protease 11 (USP11) is best known for its role in DNA damage repair by homologous recombination (HR) (11, 12). Knockdown of USP11 results in spontaneous activation of the DNA damage response and sensitivity to genotoxic stress agents (13) and ionizing radiation (14). DNA damage repair by HR mostly occurs in S and G_2_ phases of the cell cycle and is suppressed in G_1_. As part of the mechanism controlling this process, USP11 regulates the ubiquitination status of the BRCA2–PALB2 complex, and USP11 is up-regulated in S phase (12).

USP11 silencing also inhibits transforming growth factor-β pathway signaling (15), and USP11 is involved in NF-κB signaling pathways. The stability of several other important proteins relevant to health and disease is regulated by USP11, including p21 (16), RAE1 (17), and cIAP2 (18). Consistent with an important regulatory role, USP11 is dysregulated in a number of cancers, including pancreatic cancer (19), glioma, ovarian, and breast cancers, as well as hematological malignancies (12).

**Figure 1.**
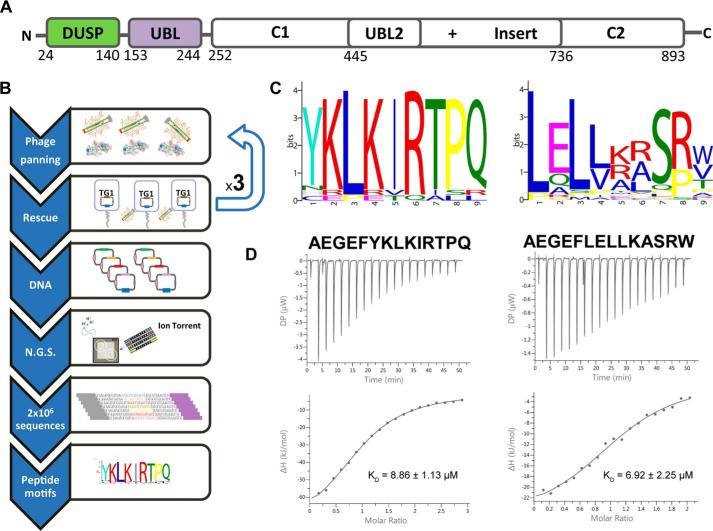
**Discovery of USP11-binding sequences.** *A,* schematic representation of the human USP11 domain structure. The N-terminal DUSP–UBL domains used as bait in the NGPD experiments (USP11_DU) are labeled and depicted in *green* and *purple*, respectively. *B,* flow chart of the NGPD approach. Three iterative rounds of phage selection (panning) against USP11_DU were carried out, and the eluted phages were bound to the target (USP11_DU) and an unrelated control protein in parallel. The phagemid vectors from the output phage isolated against the target and control proteins were isolated, and the DNA region encoding the peptides was amplified and deep-sequenced. Peptide sequences seen to be enriched against the target protein compared with the control are listed and motifs identified. *C,* amino acid sequence motifs identified by the MEME algorithm after the third round of biopanning. *D,* ITC data of USP11_DU with FYLIR (AEGEFYKLKIRTPQ) and L*X*LL (AEGEFLELLKASRW) peptides. Thermograms (*top*) and binding isotherms (*bottom*) fitted using a one binding-site model with associated *K_D_* values are shown. *DP*, differential power.

USP11 harbors a core protease domain as well as noncatalytic regions containing an N-terminal domain present in USPs (DUSP) and internal ubiquitin-like (UBL) domains (Fig. 1*A*) (20). USP11 shares this domain architecture with paralogs USP15 and USP4, which are known as the DUSP-UBL (DU) family of USPs (20–23).

We previously determined the crystal structure of the USP11 N-terminal DU domains and showed that USP11's noncatalytic domains do not directly auto-activate or inhibit the activity of USP11 (20). In contrast, the USP4 N-terminal DU domains are required for efficient turnover of the enzyme by promoting ubiquitin release (24). The USP4 and USP15 DU domains interact with E3 ligases (25) and the substrate recruitment factor SART3 (26, 27), but no interactions have yet been reported for these domains in USP11. We hypothesized that USP domains adjacent to the protease domain would be good targets for the identification of unique binding sequences as they significantly differ between USPs. Here, a next-generation phage display (NGPD) approach was applied to screen for peptide ligands against the USP11 N-terminal domains. NGPD combines the diversity of phage display libraries with the screening capability of next-generation sequencing platforms. This allows the replacement of immunoassay screening of several hundred randomly picked phage clones after conventional phage panning with the more comprehensive analysis of the sequence of potentially millions of ligand genes to determine enrichment of particular sequences that can then be selected for screening in biochemical assays. This method has been applied previously to the identification of peptides with specific binding traits (28–30). We discovered USP11-specific peptides harboring consensus motifs that do not interact with either paralog USP4 or USP15, and we identified a novel binding site in the USP11 N-terminal ubiquitin-like domain. Together, the data show that the NGPD strategy can be used to uncover highly selective ligands and novel binding sites for USPs as a basis for the development of new probes or anti-proliferative agents.

## Results

### Next-generation phage display strategy for the isolation of USP11 peptide ligands

An NGPD approach was applied to the identification of USP11 ligands as schematically depicted in Fig. 1*B*. A randomized linear peptide library fused to the gpVIII protein was used to isolate peptides that interact with the USP11 N-terminal DU domains (DUSP and UBL domains in tandem; USP11_DU; Fig. 1*A*). After three biopanning rounds, phage ssDNA was extracted and purified, and peptide sequences were amplified and deep-sequenced. To rank the identified sequences, a two-proportion Z test–based method (31, 32) was applied to round 3 output phage, and analysis of the 50 sequences with the highest Z scores after three rounds of panning (Fig. S1) revealed the presence of two sequence motifs: (YNHC)-(±)-L-(±)-φ-R (motif 1, based on 18 peptides) and a L-*X*-L-φ-*X-X*-S-(RP) (motif 2, based on 29 peptides), where *X* stands for any residue, (±) for charged residues, and φ for nonpolar residues. A graphical representation of the residue frequency within the motifs as computed by the Multiple EM for Motif Elicitation (MEME) algorithm (33) is shown in Fig. 1*C*. The thermodynamic binding parameters of synthetic peptides representative of both motifs, including the N-terminal phage protein sequence (AEGEF), AEGEFYKLKIRTPQ (referred to as FYLIR peptide) and AEGEFLELLKASRW (referred to as L*X*LL peptide), were determined by isothermal titration calorimetry (ITC). Peptides harboring either motif bound USP11_DU with dissociation constants of about 10 μm (8.86 ± 1.13 μm for AEGEFYKLKIRTPQ and 6.92 ± 2.25 μm for AEGEFLELLKASRW; Fig. 1*D*). We subsequently focused on the FYLIR peptide and showed that full-length USP11 and its N-terminal domains alone display comparable dissociation constants indicating that the presence of the protease domain has no influence on the interaction (Fig. S2).

### A helical motif in the peptide ligand binds to a novel USP11 UBL domain-binding site

**Figure 2.**
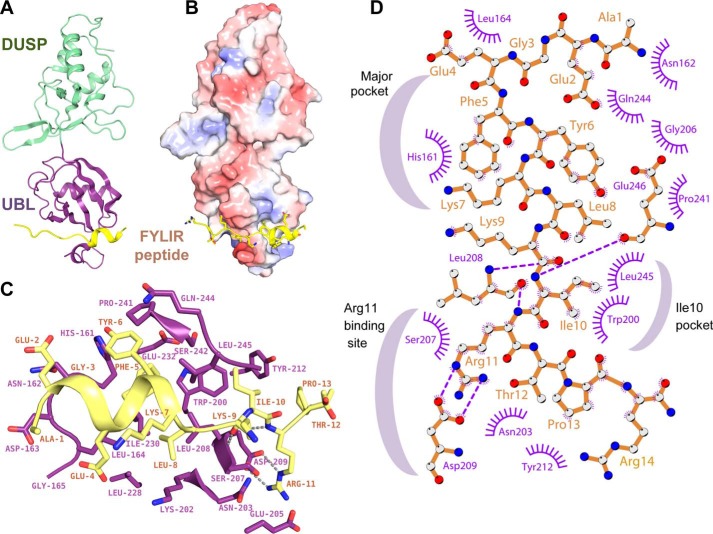
**Molecular basis of USP11–FYLIR peptide interaction.** *A, cartoon* representation of the USP11_DU–FYLIR peptide complex crystal structure. The USP11 DUSP and UBL domains are depicted in *green* and *purple*, respectively, with the FYLIR peptide (AEGEFYKLKIRTPR) shown in *yellow. B,* electrostatic potential surface representation of USP11_DU in complex with the FYLIR peptide in *yellow cartoon* representation. Side chains are shown as *sticks* in the same orientation as in *A. C,* close-up view of the molecular basis of the interactions. The peptide is bound to the USP11 UBL domain, with residues 5–12 predominantly contributing to the interaction. Key residues involved in the interaction are labeled and shown as *sticks*. Color code is the same as in *A. D,* schematic representation of FYLIR peptide–USP11 interactions generated using Ligplot+ (60). Peptide residues are labeled in *brown*, and USP11 residues are labeled in *purple*. Hydrogen bonding interactions are indicated as *purple*; *dashed lines* and USP11 residues engaging in hydrophobic interactions with the peptide are depicted in *purple. Crescent shapes* indicate USP11-binding pockets involved in FYLIR peptide binding.

At present, no binding sites or interaction partners have been identified for the USP11 N-terminal domains. We therefore focused on exploring the molecular basis of the interactions between USP11 and the peptide ligand. Co-crystallization trials of USP11_DU in the presence of the FYLIR peptide yielded monoclinic crystals of space group *P*2_1_ that diffracted to 1.3 Å resolution after optimization. The structure was determined by molecular replacement and refined to an *R*_work_/*R*_free_ of 15.7/17.8%. Data collection and refinement statistics are summarized in Table 1. The DUSP and UBL domains in the peptide complex structure are arranged in tandem (Fig. 2*A*), analogous to the structures of the rat USP11 ortholog (20) and homologs USP4 (SGC) and USP15 (21, 22), but unlike the previous structure of human USP11_DU (20). Unexpectedly, the structure revealed that the peptide occupied an elongated cleft in the USP11 UBL domain (Fig. 2), with clear electron density for peptide residues Gly-3–Arg-14 observed in both copies of the asymmetric unit (Fig. S3*A*). The peptide's main interacting core comprises nine residues (residues 5–13; FYKLKIRTP) (Fig. 2, *C* and *D*), with a buried interaction interface area of ∼750 Å^2^. Interestingly, peptide residues Glu-2–Lys-7 adopt an α-helical conformation when in complex with USP11, which is stabilized by T-shaped π–π stacking interactions of residues Phe-5 and Tyr-6. Phe-5, which can be described as the first tooth of the bidentate-like interaction, is nearly completely buried by occupying a hydrophobic pocket (referred to as major pocket). Tyr-6 is partially solvent-exposed and engages in additional contacts with the USP11 UBL domain.

**Table 1:** **Crystallographic data collection and refinement statistics**

	USP11_DU-FYLIR (AEGEFYKLKIRTPR)
**Data collection**	
Space group	P2_1_
Cell dimensions	
*a*, *b*, *c* (Å)	65.77, 45.51, 100.61
β (°)	102.68
Resolution (Å)	1.30
*R*_merge_	0.054 (0.678)*^a^*
*R*_pim_	0.027 (0.409)
*I*/σ*I*	15.1 (2.1)
*CC*1/2	0.999 (0.799)
Completeness (%)	98.3 (89.6)
Redundancy	4.1 (3.4)
Wilson B-factor (Å^2^)	13.8
**Refinement**	
Resolution range (Å)	49–1.35
No. of reflections	140,596 (13,740)
*R*_work_/*R*_free_	0.157/0.178
No. of atoms	
Protein	3986
Non-peptide ligand	38
Water	649
*B*-factors (Å^2^)	
Protein	19.66
Peptide	28.07
Water	32.36
Root mean square deviations	
Bond lengths (Å)	0.015
Bond angles (°)	1.35

*^a^* Values in parentheses are for highest-resolution shell.

The pocket is formed predominantly by side chains of USP11 residues His-161, Trp-200, Leu-208, Ile-230, Glu-232, Pro-241, Ser-242, and Leu-245. Hydrogen bonding interactions between main chain carbonyls and amine groups of peptide residues Lys-9, Arg-11, and USP11 Leu-208 are also key features of the interaction (Fig. 2, *C* and *D*). The peptide's Ile-10 occupies a second hydrophobic pocket (USP11 Leu-245, Trp-200). Ile-10 “anchors” the peptide together with the guanidinium group of FYLIR peptide Arg-11 that forms a salt bridge with USP11 Asp-209 in what could be described as the “second tooth” of the bidentate-like interaction. The peptide's Arg-11 is also in close proximity to another acidic residue, USP11 Glu-205 (Fig. 2*C*). Compared with the unliganded USP11 UBL domain (PDB code 4MEL (20)), minor conformational changes in the structure are observed. The side chain of His-161 partially masks the pocket in the unliganded structure. The loop between strands S3 and S4 in the UBL domain (S3S4 loop, residues Asn-203–Ser-207) becomes less flexible in the peptide-bound structure due to interactions of USP11 Glu-205 with the peptide's Arg-11. Moreover, a flexible-to-order transition of Leu-245 due to interactions with the peptide's Phe-5 is also observed between the unliganded and liganded USP11_DU structures (Fig. S3*B*).

### “FYLIR” peptide ligand is highly specific for USP11 in vitro

**Figure 3.**
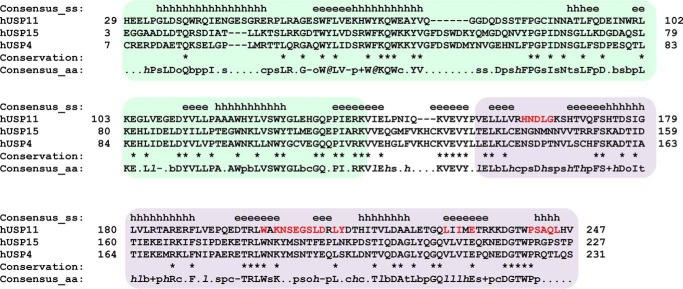
**Sequence alignment of USP11, USP4, and USP15 DU domains.** Shown is structure-based sequence alignment using PROMALS3D (61) of human USP11 (PDB code 4MEL (20)), USP15 (PDB code 3T9L (21)), and USP4 (PDB code 3JYU, Structural Genomics Consortium (SGC), J. P. Bacik, G. Avvakumov, J. R. Walker, S. Xue, and S. Dhe-Paganon, unpublished data) with secondary structure elements above the sequences indicated (*e* = strand; *h* = helix). The DUSP domain is *shaded green*, and the UBL domain is *shaded purple*. Sequence conservation is depicted as per PROMALS3D default representation (*bold uppercase letters* (such as G); aliphatic residues (I, V, L): *1*, aromatic residues (Y, H, W, F); *@*, hydrophobic residues (W, F, Y, M, L, I, V, A, C, T, H); *h*, alcohol residues (S, T); *o*, polar residues (D, E, H, K, N, Q, R, S, T); *p*, tiny residues (A, G, C, S); *t*, small residues (A, G, C, S, V, N, D, T, P); *s*, bulky residues (E, F, I, K, L, M, Q, R, W, Y), *b*, positively charged residues (K, R, H); +, negatively charged residues (D, E); −, charged (D, E, K, R, H) with the exception that identical residues are indicated using an *asterisk.* USP11 UBL domain residues located at the interface upon peptide binding are highlighted in *red. aa*, amino acid.

**Figure 4.**
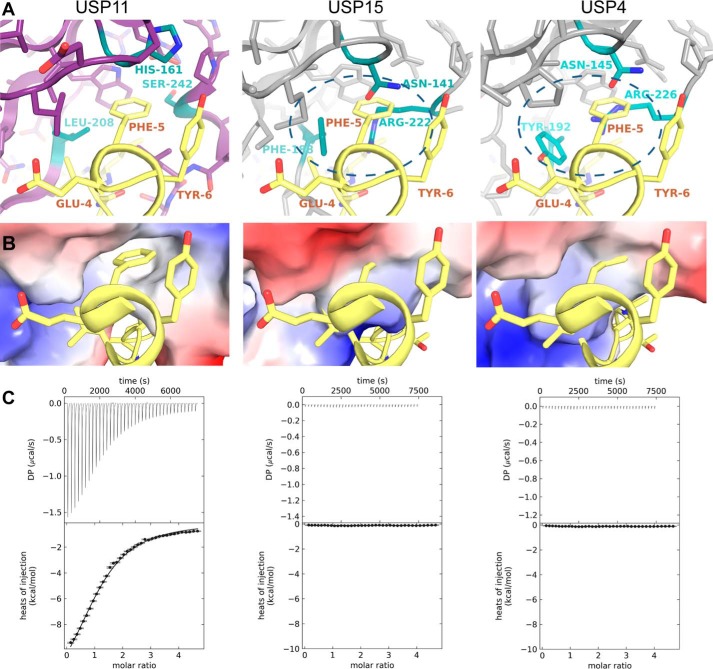
**Molecular basis of FYLIR peptide USP11 specificity.** *A,* close-up views of FYLIR peptide binding to the “major pocket” in the USP11 UBL domain (*left panel*) compared with USP15 (*center panel*) and USP4 (*right panel*), where steric clashes occur when the peptide is modeled into the same position (highlighted by a *dashed ellipse*). Key residues involved in the interaction (USP11) or preventing peptide binding (USP15 and USP4) are depicted in *cyan* and labeled. *B,* close-up views of electrostatic surface representations of FYLIR peptide binding to the major pocket in the USP11 UBL domain (*left panel*) compared with USP15 (*center panel*) and USP4 (*right panel*), where the binding pocket is occluded. *C,* ITC data of FYLIR peptide with USP11_DU, USP4_DU, and USP15_DU, showing that peptide-ligand binding is highly specific for USP11.

USP11 shares its domain structure with USP4 and USP15 (20–24). The USP11 UBL domain shares 32% sequence identity with the corresponding UBL domains in USP4 and USP15, whereas these domains in USP4 and USP15 share 72% sequence identity (Fig. 3). We therefore tested whether the peptide ligand is specific for USP11. Interestingly, an interaction between FYLIR peptide and the DU domains of either USP4 or USP15 by ITC was not observed, suggesting that this binding cleft for the peptide is specific to USP11 (Fig. 4*C*). A superposition of the three structures revealed differences in the main USP11-binding pocket (“major pocket”) compared with USP15 and USP4 (Fig. 4, *A* and *B*). In particular, USP11 Leu-208 is substituted by Phe-188 and Tyr-192 in USP15 and USP4, respectively. At the position of USP11 Ser-242, USP15 and USP4 harbor arginine residues Arg-222 and Arg-226, respectively (Figs. 3 and 4*A*). The arginine residues are engaged in π-stacking and ion–π interactions with the aromatic side chains of USP15 Phe-188 and USP4 Tyr-192 and consequently occlude the pocket. Therefore, the smaller side chains of Leu-208 and Ser-242 may be the key residues that allow USP11 to engage with the peptide ligand.

**Figure 5.**
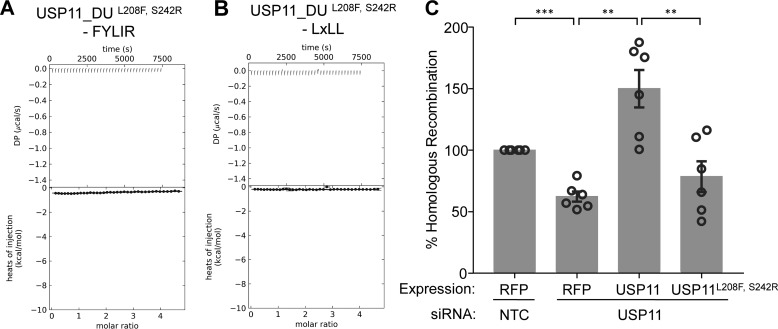
**Effects of a UBL-binding site mutant on peptide recognition and homologous recombination.** *A,* ITC data of the USP11_DU^L208F/S242R^ double mutant that mimics USP15 showing that binding of the FYLIR peptide ligand is completely abolished upon mutating residues in the UBL pocket (referred to as major pocket). *B,* ITC data of titrations of peptide AEGEFLRLLNFTKP harboring motif 2 (L*X*LL) with USP11_DU^L208F/S242R^. The interaction of this peptide ligand with USP11 is also abolished upon mutating residues in the UBL pocket. *C,* homologous recombination GFP-reporter assays with USP11 siRNA and RFP-USP11 WT or mutant USP11^L208F/S242R^. Each individual experiment contains three technical repeats. Data presented are the overall mean calculated from the means of each individual experiment (*n* = 6). % GFP and RFP double-positive cells were normalized to RFP transfection efficiency; *error bars* = S.E.; *p* values were computed using the Welch's *t* test and are shown as ***, *p* < 0.0005; **, *p* < 0.005 (NTC *versus* siUSP11, *p* = 0.0003; siUSP11 *versus* siUSP11 + WT, *p* = 0.0017; and siUSP11 *versus* siUSP11 + USP11^L208F/S242R^, *p* = 0.0049).

To further verify the key role of this pocket for ligand binding in solution, we generated a USP11_DU L208F and S242R double mutant (USP11_DU^L208F/S242R^) to render this region USP15-like. USP11_DU and USP11_DU^L208F/S242R^ behaved similarly on gel filtration. No aggregation and an equivalent elution volume were observed for the mutant disclosing that the mutations are compatible with folding resulting in a similar hydrodynamic radius to USP11_DU. Furthermore, FL-USP11^L208F/S242R^ was active (Fig. S4). ITC experiments confirmed that ligand binding to USP11_DU^L208F/S242R^ was abolished (Fig. 5*A*). We also investigated whether this binding pocket is involved in interactions with motif 2 (L*X*LL peptide). ITC experiments with the USP11 pocket-deficient double mutant USP11_DU^L208F/S242R^, and this peptide resulted in no detectable binding, showing that both peptide-binding motifs require the major pocket for interaction with USP11 (Fig. 5*B*). Whether in addition to altering the pocket region these mutations affect the conformation of FL-USP11 is not known.

### UBL-binding pocket of USP11 is required for its function in homologous recombination

The ITC data showed that peptides harboring either motif interacted with the major pocket in the UBL domain. USP11 is most well-known for its role in DNA damage repair by HR (11, 12), and depletion of USP11 results in HR repair defects (13, 14). To explore whether the identified binding site is required for USP11 function, HR GFP-reporter assays were carried out in U20S-DR3 reporter cells (34). These cells contain an integrated nonfunctional GFP gene, which has been interrupted by inclusion of an I-Sce1 restriction site. Also contained within the integrated region is another incomplete GFP sequence that can be used as a template for HR repair across the I-Sce1 site. A double-strand break within the reporter can be induced by expression of the nuclease I-Sce1 and reconstitution of the GFP gene and subsequent expression of functional GFP protein can be used as a marker of successful HR-mediated repair. To this end, the formation of GFP products was measured from the integrated substrate in USP11-depleted cells transfected with I-Sce1 (34) and complemented with USP11WT or the UBL-binding site mutant USP11^L208F/S242R^ (Fig. 5*C*). The percentage of GFP-positive cells normalized to the RFP transfection efficiency was significantly lower in the case of the USP11-binding site mutant compared with USP11WT and similar to the RFP control. This shows that the USP11^L208F/S242R^ mutant cannot complement USP11WT for HR indicating the importance of this binding site for USP11's function in HR, either directly or due to its contribution to the conformation of the enzyme.

### Transduction of a “FYLIR” peptide agent results in differential effects on cell viability

It was hypothesized that peptide binding may affect the catalytic turnover of substrates by steric hindrance as the peptide-binding site is located in proximity to the catalytic triad in the protease domain. When the impact of the FYLIR peptide on the USP11 catalytic activity was assessed using the model substrates 7-amino-4-methylcoumarin-modified ubiquitin and Lys-63–linked FRET di-ubiquitin carboxytetramethylrhodamine (TAMRA), the observed slight reduction in catalytic activity was not statistically significant compared with controls.^7^

**Figure 6.**
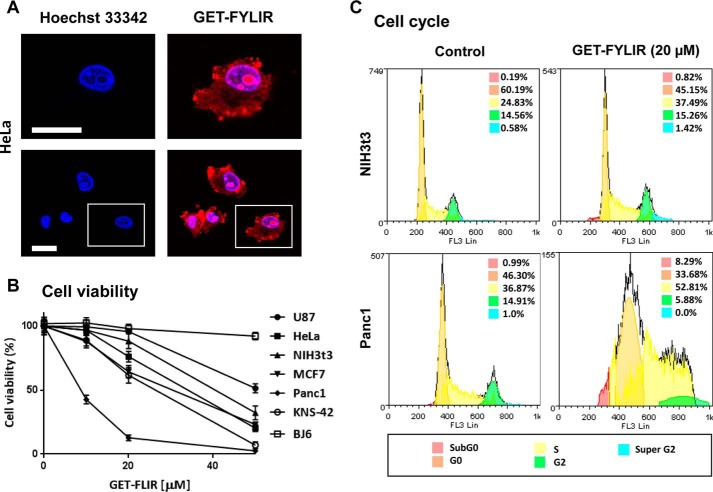
**Cellular effects of FYLIR peptide agent.** *A,* GET-FYLIR has cytoplasmic, nuclear, and nucleolar localization. HeLa cells were treated with GET-FYLIR (10 μm) for 24 h and assessed by confocal microscopy (nuclei were counterstained with Hoechst 33342). The *white scale bar* is 20 μm. *B,* GET-FYLIR decreases cell viability cell type–specifically and dose-dependently. Cell viability (Presto Blue assay) was assessed after 24 h of incubation with GET-FYLIR (0, 10, 20, and 50 μm) in KNS-42, U87, BJ6, HeLa, NIH3T3, MCF7, and Panc1 cells. Cell viability was expressed as percentage of cell viability ± S.D. (*n* = 3 biological repeats). *C,* GET-FYLIR induces S phase arrest cell type–specifically. Cell cycle analysis was conducted using NIH3T3 cells (*n* = 5 biological repeats) and Panc1 cells (*n* = 7 biological repeats) untreated or incubated with GET-FYLIR (20 μm) for 24 h. Cells were fixed, stained with propidium iodide staining solution, and analyzed for DNA content. The distribution and percentage of cells in subG_0_, G_0_, S, G_2_, and super-G_2_ phase of the cell cycle are indicated.

We next evaluated the effect of the FYLIR peptide ligand on cells in culture. The novel GET system, developed for highly efficient nucleic acid, protein and peptide delivery (35), was used to deliver the FYLIR peptide. GET is based on the use of cell-penetrating peptides modified with heparin-binding sequences, which enhance cellular uptake by endocytosis. The synthetic peptide, GET-FYLIR, designed for these studies harbored an N-terminal TAMRA fluorescent tag, for easy visualization, fused to the GET (P21 and 8 Arg (35)) and FYLIR sequences. GET-FYLIR was efficiently delivered to all cell lines tested (including human glioblastoma KNS-42 and U87, cervical carcinoma HeLa, mammary carcinoma MCF7, pancreatic carcinoma Panc1, and fibroblast NIH3T3 and BJ6 cell lines). Confocal imaging of HeLa cells, treated for 24 h with a low dose of GET-FYLIR (10 μm), showed localization of the GET-FYLIR peptide in both the cell cytoplasm and the nucleus, with the highest accumulation in nucleoli (Fig. 6*A*). This showed that the GET-FYLIR peptide is efficiently delivered to subcellular compartments where endogenous USP11 protein is localized (36) and that the GET-FYLIR peptide efficiently escapes endosomal vesicles (as endosomal escape can be a limitation for delivered peptides). Having established efficient delivery, the peptide's effect was investigated by assessing cell viability.

Interestingly, a significant cell type-specific decrease in cell viability upon delivery of the GET-FYLIR peptide (up to 50 μm) was observed (Fig. 6*B*). Panc1 cell lines were particularly sensitive to GET-FYLIR dose, whereas other cell lines, especially BJ6 human dermal fibroblasts, were relatively insensitive. After 24 h of incubation with 10 μm GET-FYLIR, Panc1 cells showed a loss of viability (∼50%); an almost complete loss of viability (to ∼12%) was observed at a dose of 20 μm. Viability of BJ6, U87, and NIH3T3 cell lines was retained at a 10 μm dose with a minimal effect at a 20 μm dose. To confirm that the differential effect on cell viability was not due to peptide uptake efficiency, GET-FYLIR initial uptake (1 h) and long-term uptake (24 h) with low dosages in NIH3T3 cells (a relatively insensitive cell line) were compared with uptake in Panc1 cells (a sensitive cell line to GET-FYLIR dose) (Fig. S5). The lowest dose tested (1 μm; Fig. S5*A*) showed little difference in cell uptake in either cell type for short (1 h) or long (24 h) exposures. Importantly, uptake increased with longer exposures. However, at a higher dose (5 μm; Fig. S5*B*), cell viability effects in Panc1 but not NIH3T3 cells were observed, with increased uptake in Panc1 cells for both 1- and 24-h incubations. Interestingly, the uptake was similar at 1- and 24-h incubations for Panc1 cells at this higher dose. This possibly suggests that cell membrane permeability may have been affected even after a 1-h incubation in Panc1 cells. Uptake by NIH3T3 cells was significantly lower than Panc1 cells at this dose for both incubation times. These data suggest that the GET-FYLIR peptide is similarly taken up in the different cell lines, but that the uptake is significantly altered once the concentration begins to compromise cell viability. To confirm that GET-FYLIR was specifically producing this change in cell viability and uptake, a scrambled control peptide where the FYLIR sequence was replaced by the sequence APKFEIRRGTYKLE (GET-Scrambled) was also used. The scrambled peptide was efficiently delivered, and there was no difference in the uptake profile between NIH3T3 and Panc1 cells. Furthermore, the GET-Scrambled peptide had no significant effect on Panc1 cells (up to 10 μm) (Fig. S6). This therefore suggests that cell type–specific changes in cell uptake, at dosages in which the cell viability was compromised, were attributable to the activity of the FYLIR peptide.

To further characterize the effect of the GET-FYLIR peptide, cell cycle analysis was conducted on cell lines that were GET-FYLIR–sensitive (Panc1) and relatively resistant (NIH3T3). Using genome copy number analyses by flow cytometry, we demonstrated that Panc1 cells treated with GET-FYLIR (20 μm) showed a significantly increased fraction of cells in S phase (36.9–52.8%; *p* < 0.0001; *n* = 7), as well as significant alterations in all other fractions (Fig. 6*C*). In NIH3T3 cells, GET-FYLIR had smaller effects on cell cycle distribution (24.8–37.5%, *p* < 0.0001, *n* = 5). Furthermore, in Panc1 cells, the subG_0_ fraction (cells containing less than one diploid genome) increased ∼9-fold (0.9–8%; *p* < 0.0001; *n* = 7), whereas this effect was not significant in NIH3T3 cells (0.2–0.8%: *p* = 0.9992: *n* = 5), suggesting DNA fragmentation and cell death is triggered in Panc1 cells by GET-FYLIR. These results suggest that, when efficiently delivered to cells, FYLIR has a cell type–specific effect on cell viability, associated with cell cycle arrest and S phase progression.

## Discussion

This study describes the discovery for the first time of unique peptide ligands for a ubiquitin-specific protease by the means of next-generation phage display. The highest affinity peptide with the “FYLIR” motif interacts with USP11 with a dissociation constant of ∼10 μm. This ligand is highly specific for USP11 by occupying an unexpected unique site in the N-terminal UBL domain that is neither present in the paralogs, USP4 and USP15, or any of the other USPs encoded in the human genome. UBL domains are often involved in interactions and occur frequently in USPs (37). In the most well-studied member of the USP family USP7, UBL domains recognize binding motifs in the epigenetic regulators UHRF1 (38) and DNMT1 (39) among other binding partner proteins (40), but this is achieved through different recognition surfaces. The USP11 crystal structure showed that both the S3–S4 loop that is often involved in engaging binding partners in UBL domains (41) and a conserved unique C-terminal sequence extension to the UBL core (sequence WPSAQL) are part of the peptide-binding site.

A single- to double-digit micromolar starting affinity, as reported here, is often observed for peptide identification from phage display. For example, peptides isolated to Japanese encephalitis virus, insulin-degrading enzyme, or IL17A had affinities in the 2.5–12 μm range (42–44). Subsequent affinity maturation has been successfully employed to achieve affinities in the nanomolar range (42). Additional peptide phage libraries can also be constructed by randomizing the residues that are adjacent to the ones critical for the interaction (soft randomization) based on structural information and thus improve the affinity of the parental FYLIR peptide. This will be the focus of future investigations.

For the majority of USPs, the roles of noncatalytic regions, such as the N-terminal USP11_DU domains, are poorly understood. However, USP7 accessory domains have been shown to have unique specificities, where (PA)*XX*S motifs in p53, Hdm2, and Epstein-Barr nuclear antigen 1 are recognized by a central binding cleft in the N-terminal TRAF-like domain (45) and the USP8 rhodanese domain harbors a ligase recognition site (46). Knowledge of binding sites and the availability of agents targeting the noncatalytic domains can be beneficial for drug discovery programs due to the potential for increased selectivity compared with active-site inhibitors. This is the first report of specific binding sequence motifs for USP11. A high-resolution crystal structure revealed a binding mode with a helical segment that bears hallmarks of typical peptide–protein interactions such as an amphiphilic nature and the presence of a leucine, tyrosine, isoleucine, and phenylalanine at the interface (47). Interestingly, both peptide motifs identified in this unbiased NGPD approach use the major pocket for interactions. In addition, a role of this binding site in homologous recombination assays was confirmed by comparing USP11WT and the binding pocket–deficient mutant USP11^L208F/S242R^. This may be due to a direct contribution of the particular mutated residues to interactions, but it could, in principle, also be due to a change in enzyme conformation.

Furthermore, a fluorescently labeled peptide agent was designed and showed efficient transduction of several cell lines. Interestingly, the FYLIR peptide-based agent blocked cell proliferation and affected viability by arresting the cell cycle predominantly in S phase. USP11's role in DNA damage repair by homologous recombination, which is important in S phase and is highly suppressed in G_1_ (12), is consistent with the peptide's effects being, at least in part, attributed to interference with USP11 function. Indeed, mutation of the FYLIR peptide's USP11-binding site affected homologous recombination in U2OS cells. A significant fraction of USP11 localizes to chromatin in cells before and upon induction of DNA damage (13). The GET-FYLIR peptide localizes with the USP11 subcellular distribution and is enriched in nucleoli.

Several reports link USP11 to cell cycle progression and proliferation, although the detailed mechanisms are only partially understood. For example, (i) cell growth assays in MCF-7 and MDA-MB-231 cells show an increase in cell numbers upon USP11 overexpression, whereas USP11 knockdown leads to growth inhibition (48); (ii) USP11 itself is under cell cycle control and turns over rapidly in G_1_ cells, especially upon DNA damage induction, whereas expression of USP11 in S phase is high and insensitive to DNA damage (12). Knockdown of USP11 results in the activation of DNA damage-response pathways, leading to hypersensitivity of cells to genotoxic stress (13); (iii) in primary human fibroblasts knockdown of USP11 results in characteristics of senescence, including proliferative arrest and enlarged nucleoli (49); and (iv) USP11 interacts with p21 to regulate cell cycle progression and DNA damage responses (16). Interestingly, we found that in Panc1 cells that have previously been shown to have high amounts of USP11 mRNA and to undergo dose-dependent cell death upon treatment with mitoxantrone that inhibits USP11 (19), cell viability is highly affected by FYLIR peptide exposure. USP11 has been recognized as a novel anti-cancer target by preventing repair of double-strand breaks in cancer cells via synthetic lethality (11). Nevertheless, further studies will be required to decipher the detailed mechanism of action of the GET-FYLIR peptide agent.

Taken together, we identified USP11 interaction motifs, a novel binding site in the USP11 UBL domain, and demonstrated the ability of a peptide agent containing a USP11 interaction motif to differentially affect cell lines. Therefore, as well as providing insight into USP11 recognition, the findings may also pave the way for the development of novel peptidomimetic agents. To our knowledge, outside of epitope mapping, this is the first example of using NGPD to define protein-binding motifs. The strategy will be applicable to other members of the USP family for the identification of unique ligands and binding sites and the development of molecular probes and/or therapeutic agents.

## Experimental procedures

### Constructs

DNA constructs for this study were generated by standard molecular biological techniques. Constructs of human USP11 spanning the DUSP–UBL domains (USP11_DU), residues 1–244 or 24–244, USP11(1–920), as well as USP15_DU and USP4_DU in pET26b and FL-USP11 in pColdI have previously been described (20, 21). The double mutants USP11_DU^L208F/S242R^ and FL-USP11^L208F/S242R^ were generated by site-directed mutagenesis using the QuikChange method. For HR assays, FL-USP11 was cloned into the cDNA3.1(+)mRFP vector using KpnI and XhoI restriction sites and a corresponding construct harboring L208F and S242R mutations created by the QuikChange method.

### Protein production

All proteins were expressed and purified according to protocols described previously (20). Protein expression was induced by adding 0.5 mm isopropyl 1-thio-β-d-galactopyranoside to BL21-CodonPlus cell cultures grown at 37 °C in 2× YT medium to mid-log phase. Cells harboring the USP11_DU (residues 1–244 or 24–244), the double mutant USP11_DU^L208F/S242R^, USP15_DU, and USP4_DU plasmids were harvested after 4 h. For FL-USP11 constructs, cells were grown for 72 h at 10 °C. For all DU constructs, cells were lysed by sonication in 20 or 50 mm Tris-Cl, pH 7.5, 150 mm NaCl, 20 mm imidazole. Cells containing FL-USP11 were lysed in 50 mm Tris-Cl, 300 mm NaCl, 5% (v/v) glycerol, 20 mm imidazole. Samples were purified using HiTrap chelating columns (GE Healthcare) precharged with nickel sulfate. Size-exclusion chromatography was performed using a Superdex 75 16/60 column (GE Healthcare) pre-equilibrated with 50 mm Tris-Cl, pH 7.5, and 150 mm NaCl for the DU constructs or a Superdex 200 16/60 column (GE Healthcare), pre-equilibrated with 50 mm Tris-Cl, pH 7.5, 300 mm NaCl, and 1% glycerol for the FL-USP11.

### Phage display and biopanning

The peptide phage display library used for biopanning experiments (kindly provided by Professor Franco Felici, University of Molise, Italy; designated PC89VIII) is based on the PC89 phagemid vector (50) with a nonapeptide inserted at the N terminus of the phage major coat protein pVIII and a reported diversity of ∼10^7^. The library was transformed into *Escherichia coli* TG1 supE thi-1 Δ(lac-proAB) Δ(mcrB-hsdSM)5(rK-mK) [F′traD36 proAB lacIqZΔM15], which was the strain used for all manipulations and phage rescue. Libraries were grown to mid-log phase (OD_600_ ∼0.6), superinfected with M13KO7 helper phage (Pharmacia LKB), and incubated overnight at 30 °C (250 rpm) to produce phage particles. Phage was harvested, and PEG was precipitated only for the first panning round. For all subsequent selection rounds (three in total), supernatant phage was used directly. Biopanning was carried out once, as described previously (51), and proteins were immobilized on cobalt beads (Dynabeads) (solution phase panning). A total of 100 μg of USP11_DU was immobilized for each round. In parallel, the phage isolated at each round against USP11_DU was also bound against an unrelated His-tagged control protein under the same conditions. This control protein was a fragment of flagellin, a virulence factor from *E. coli* O157:H7, amino acid residues 164–495, and the protein was used as it is unrelated to the target proteins and carried a C-terminal His tag. Bound phage were washed 10–20 times with PBS (10 mm phosphate buffer, pH 7.2–7.4, with 150 mm NaCl) plus 0.1% (v/v) Tween (PBST) and then PBS. Bound phage were eluted from the beads with 100 mm triethylamine and neutralized in 1 m Tris, pH 7.4. Half of the eluted phage was used for infection of TG1 cells, which were plated and used to make glycerol stocks.

### Deep sequencing

The ssDNA of eluted phage for round 3 of selection against the USP11_DU target and the control protein was extracted and purified using the QIAprep Spin M13 kit. Subsequently, phage ssDNA was precipitated with ethanol and stored as a dry pellet to be used as a PCR template. For the amplification of the unknown peptide sequences, a two-step PCR strategy was followed, as described previously (32). The strategy produced an amplicon of ∼330 bp, and each sample contained a unique barcoded sequence. Each sample's DNA concentration was determined by the Agilent 2100 Bioanalyzer using the DNA 1000 kit. Based on the concentration readings for the peak of interest, samples were pooled in equal amounts, purified by the Agencourt AMPure XP kit, and run on a 2% (w/v) agarose gel. The band of ∼330 bp was excised from the gel and purified using the NucleoSpin Gel and PCR Clean-up kit. Finally, the concentration and size of the pooled DNA were measured on a Bioanalyzer. NGPD was carried out once for each round of panning.

### Ion torrent data file processing and sorting of sequences

Deep sequencing of all samples was performed using an Ion Torrent PGM technology commercial service by the Department of Biochemistry at the University of Cambridge on a 318 chip. Approximately 500 Mbase of readings were obtained per chip, which translates to ∼10^5^ reads on average per barcode and a mean length of 100 bp. The number of meaningful reads per panning round for each sample was found to be ∼4 × 10^4^. Subsequently, processing of all sequences in quality FASTQ file format was conducted. The first processing step consisted of the demultiplexing of the FASTQ file to sort the sequences according to their barcodes and to generate individual FASTQ files, each representing the repertoire of peptides eluted from one of the panning rounds binding to either the target protein or the control protein. Each FASTQ file was then converted to FASTA format, translated in all three reading frames, and concatenated into one file containing all frames. Using Perl scripts, only sequences of interest, flanked between conserved PC89 gpVIII protein sequences (AEGEF and DPAKAA motifs were used), were kept in a single file. The peptide sequence repertoires of eluted polyclonal phage binding to either the protein of interest or the control protein were then compared. Ultimately, all sequences were exported to excel files with frequencies: the number of copies of the sequence isolated against the target protein and the number of copies of the same sequence isolated against the control protein. A two-proportion Z test was then used to compare the sequence populations and sort them by Z scores, which reflects the ratio as well as the absolute frequencies of each peptide sequence and allows their ranking by relative statistical importance (31). For peptide motif generation from the obtained sequences, the MEME algorithm was used (33).

### ITC

ITC data were measured on a MicroCal VP-ITC or a PEAQ ITC instrument to obtain the binding parameters for peptide ligand–USP11_DU interactions. The sample cell contained 30 μm protein in PBS buffer. Each peptide (at a concentration of 300 or 600 μm) was titrated into the sample cell in 8-μl (VP-ITC) or 2-μl (PEAK ITC) injections at a temperature of 25 °C. Spacing was typically 180 s, and a stirring speed of 300 rpm was used. The data were analyzed using NITPIC (52), SEDPHAT (53), and GUSSI software. ITC experiments were repeated at least twice independently apart from the USP15_DU, USP4_DU, and the FL-USP11 titrations with FYLIR peptide, which were conducted once.

### Protein crystallization, data collection, and structure determination

Samples of the USP11 DUSP–UBL domains (residues 24–244) at concentrations of 4 and 8 mg/ml in 100 mm NaCl, 50 mm Tris-Cl, pH 7.5, 1% glycerol were mixed with the FYLIR peptide (AEGEFYKLKIRTPR) at a ratio 1:4, and crystallization trays were set up by the sitting drop vapor diffusion method at 10 °C. Crystals grew within 3 days, and the initial conditions were further optimized in separate trays varying the pH and the precipitant concentration. In 0.01 m tri-sodium citrate and 16% PEG6000 rectangular single crystals grew within a day. Crystals were flash-cooled after soaking in a cryoprotectant solution of 0.01 m tri-sodium citrate, 16% PEG6000, 20% glycerol, and 10% ethanediol. Crystals of the USP11_DU–FYLIR complex diffracted to 1.3 Å resolution, and a dataset was collected at beamline I02 at Diamond Light Source, UK, at a wavelength of 0.97949 Å and a temperature of 100 K. Data were processed using XDS (54) and AIMLESS (55), and the structure was solved by molecular replacement using individual DUSP and UBL domain coordinates from the human USP11_DU structure at 3.0 Å (PDB code 4MEL (20)) as search model with PHASER (56). Data collection statistics are summarized in Table 1.

### Model building, refinement, and validation

Model building and adjustments of the two molecules of the asymmetric unit, each binding to a peptide at the same location, were conducted using COOT (57). Structure refinement was carried out in PHENIX (58); data refinement statistics are shown in Table 1. The quality of the model was assessed by MOLPROBITY (59). The final model consists of two USP11_DU molecules in the asymmetric unit that associate via an interface area of 812 Å^2^, but no tight packing at the interface occurs. Either 12 or all 14 residues of the FYLIR peptide were observed in each copy of the asymmetric unit. LigPlot^+^ (60) was used to analyze the interactions of peptide residues with USP11_DU. In the final model, there are no Ramachandran outliers with 98.7% of residues located in favored regions. Refinement statistics are summarized in Table 1. All structure figures were generated with PyMOL.

### Cell lines

Cell lines (human glioblastoma KNS-42 and U87, cervical carcinoma HeLa, mammary carcinoma MCF7, pancreatic carcinoma Panc1, and fibroblast NIH3T3 and BJ6 cell lines) except KNS-42 were cultured at 37 °C in 5% CO_2_ in Dulbecco's modified Eagle's media (DMEM; Sigma), supplemented with 10% (v/v) fetal calf serum (Sigma), 4.5 g/liter d-glucose, 2 mm l-glutamine, 100 units/ml penicillin, and 100 units/ml streptomycin (Invitrogen). KNS-42 cells were cultured in DMEM/F-12 (1:1). Cell passage was carried out using 0.05% (w/v) trypsin/EDTA (Invitrogen). U20S-DR3-GFP reporter cell lines for homologous recombination were a kind gift from Jeremy Stark (City of Hope, Duarte, CA).

### DNA repair reporter assays

U20S reporter cell lines were simultaneously co-transfected with siRNA using Dharmafect1 (Dharmacon) and DNA (RFP-USP11 or RFP-USP11^L208F/S242R^ and ISce1 endonuclease expression constructs) using FuGENE6 (Promega). The media were replaced after 16 h, and cells were grown for a further 48 h before harvesting with trypsin and fixation in 2% paraformaldehyde (Sigma) in PBS. Using a CyAn flow cytometer, a minimum of 10,000 cells was analyzed for each sample, and RFP and GFP double-positive cells were scored and normalized to RFP-transfection efficiency. Each individual experiment contained three technical repeats. Graphs shown are combined data from six independent experiments, and error bars show S.E.

### GET-FYLIR peptides

Synthetic peptides were obtained from Biomatik or PeptideSynthetics. These harbored an N-terminal red-fluorescent TAMRA–modified GET sequence composed of P21 (KRKKKGKGLGKKRDPCLRKYK) and 8R (RRRRRRRR) fused to the FYLIR peptide (AEGEFYKLKIRTPR) to create GET-FYLIR (TAMRA-KRKKKGKGLGKKRDPCLRKYKRRRRRRRRAEGEFYKLKIRTPR-NH_2_). In the equivalent scrambled GET control peptide the FYLIR sequence was replaced by the scrambled sequence APKFEIRRGTYKLE (GET-Scrambled).

### Cell uptake

Cells treated with GET-FYLIR or GET-Scrambled were analyzed on an Astrios cell sorter using a 488-nm green laser (40,000 cells; gated on live cells by forward/side scatter). Gmean was used as the average cell intensity. All data sets were combined for the statistical analysis. A Sidak's multiple comparisons test was applied. *N* refers to the number of biological replicates.

### Confocal microscopy

HeLa cells were plated onto 10-mm diameter glass coverslips (1 × 10^5^ cells/coverslip). Cells treated with GET-FYLIR were fixed for 10 min in 3.7% (w/v) paraformaldehyde (Sigma), stained with 1 μg/ml Hoechst 33342 (Molecular Probes) for a further 10 min, washed, and mounted. Cells were imaged using an LSM880C confocal microscope (Zeiss, Germany). A ×63 objective lens was used with a 488-nm laser used for Hoechst 33342 and a 561-nm DPSS laser for GET-FYLIR–transduced cells. Images were captured and processed using ZEN software (Zeiss, Germany).

### Cell viability

Cells were plated in 96-well plates (2.5 × 10^4^ cells/well) and treated with GET-FYLIR (0–50 μm) for 24 h. Cells were also treated with GET-Scrambled as a control. After treatment, cells were washed with PBS and then incubated for 15 min at 37 °C with a 100-μl Presto Blue solution (10% Presto Blue, Invitrogen in Hanks' balanced salt solution, Sigma). Change in the fluorescence was measured using a plate reader with the excitation/emission wavelengths set at 560/590 nm (Infinite® 200 PRO, TECAN), with untreated cells used as a control. All data sets were combined for the statistical analysis. A Sidak's multiple comparisons test was applied. *N* refers to the number of biological replicates.

### Cell cycle analysis

Cells were plated in 24-well plates (1 × 10^5^ cells/well) and treated with GET-FYLIR (20 μm) for 24 h. The cells were treated with trypsin, detached from the plate, and centrifuged at 200 × *g* for 5 min. The supernatant was aspirated, and the cells were resuspended in PBS. The cells were fixed in 70% ethanol for 1 h at 4 °C. The cells were washed with PBS and incubated in propidium iodide staining solution (0.1% (v/v) Triton X-100, 10 μg/ml propidium iodide (Sigma), and 100 μg/ml DNase-free RNase A in PBS) for 30 min at room temperature. Cells were analyzed using an FC500 flow cytometer (Beckman Coulter) equipped with a 488-nm laser, and data from at least 10,000 cells were acquired for each sample. Cells were gated based on forward and side scatter, and doublets excluded by height/area analysis. Cell cycle percentages were calculated by curved fit using Weasel Version 3.0 (Walter and Eliza Hall Institute of Medical Research). Data shown are representative. All data sets were combined for the statistical analysis. A Sidak's multiple comparisons test was applied. *N* refers to the number of biological replicates. All curves and statistical analyses were produced using Prism 7 (GraphPad Software).

## Author contributions

A. S., L. B. F., R. M. D., B. C. M., J. R. M., J. E. D., K. C. G., and I. D. formal analysis; A. S. and S. G. C. validation; A. S., L. B. F., R. M. D., and S. G. C. investigation; A. S., L. B. F., R. M. D., J. E. D., and I. D. visualization; A. S., L. B. F., K. C. G., and I. D. writing-original draft; B. C. M. resources; J. R. M., J. E. D., K. C. G., and I. D. supervision; K. C. G. and I. D. project administration; I. D. conceptualization; I. D. funding acquisition.

## Supplementary Material

Supporting Information

## Footnotes

7 S. G. Caulton and A. Spiliotopoulos, unpublished observations.

## Footnotes

6 The abbreviations used are:

USP: ubiquitin-specific protease
NGPD: next-generation phage display
ITC: isothermal titration calorimetry
UBL: ubiquitin-like
DUSP: domain present in USP
RFP: red fluorescent protein
DMEM: Dulbecco's modified Eagle's medium
ssDNA: single-stranded DNA
TAMRA: 5-carboxytetramethylrhodamine
DU: DUSP–UBL
PDB: Protein Data Bank
HR: homologous recombination.
