# Supplementary material for: Discovery of peptide ligands targeting a specific ubiquitin-like domain–binding site in the deubiquitinase USP11
Source: J Biol Chem. 2018 Oct 29;294(2):424–36. doi: 10.1074/jbc.RA118.004469 (PMC6333900; doi:10.1074/jbc.RA118.004469)
Supplement: Supporting Information [file supp_294_2_424__index.html]

Discovery of peptide ligands targeting a specific ubiquitin-like domain-binding site in the deubiquitinase USP11 — USP11 selective peptide ligands — Discovery of peptide ligands targeting a specific ubiquitin-like domain–binding site in the deubiquitinase USP11 — USP11 selective peptide ligands — Supporting Information 

# Discovery of peptide ligands targeting a specific ubiquitin-like domain–binding site in the deubiquitinase USP11

## Supporting Information

- Supporting information - Figures: S1-S6
