# Supplementary material for: Discovery of peptide ligands targeting a specific ubiquitin-like domain–binding site in the deubiquitinase USP11
Source: J Biol Chem. 2018 Oct 29;294(2):424–36. doi: 10.1074/jbc.RA118.004469 (PMC6333900; doi:10.1074/jbc.RA118.004469)
Supplement: Supporting Information [file supp_RA118.004469_138820_1_supp_216001_pgl6lv.pdf]

SUPPORTING INFORMATION

Discovery of peptide ligands targeting a specific ubiquitin-like domain-binding site in the deubiquitinase USP11

Anastasios Spiliotopoulos[1,2#], Lia Blokpoel Ferreras[1], Ruth M. Densham[3], Simon G. Caulton[1], Ben C. Maddison[4], Joanna R. Morris[3], James E. Dixon[1], Kevin C. Gough[2*] and Ingrid Dreveny[1*]

[1] Centre for Biomolecular Sciences, School of Pharmacy, University of Nottingham, Nottingham NG7 2RD, United Kingdom; [2] School of Veterinary Medicine and Science, Sutton Bonington Campus, College Road, Sutton Bonington, Leicestershire, LE12 5RD, UK; [3] Birmingham Centre for Genome Biology and Institute of Cancer and Genomic Sciences, Medical and Dental Schools, University of Birmingham, Birmingham B15 2TT, UK; [4]ADAS, School of Veterinary Medicine and Science, Bonington Campus, College Road, Sutton Bonington, Leicestershire, LE12 5RD, UK.

Running title: *USP11 selective peptide ligands*

[#]Present address: UCB Pharma, 208 Bath Rd, Slough SL1 3WE, UK.

[*]To whom correspondence should be addressed: Dr Kevin Gough, School of Veterinary Medicine and Science, Sutton Bonington Campus, College Road, Sutton Bonington, Leicestershire, LE12 5RD. UK. kevin.gough@nottingham.ac.uk, Dr Ingrid Dreveny, Centre for Biomolecular Sciences, School of Pharmacy, University of Nottingham, Nottingham NG7 2RD United Kingdom. ingrid.dreveny@nottingham.ac.uk

Contents:

Supporting Figures

- Figure S1
- Figure S2
- Figure S3
- Figure S4
- Figure S5
- Figure S6

Rank	Sequence	Z score
1	YKLKIRTPQ	(168.4)
11	YKLKVRTPQ	(5.7)
10	YKLRIRTPQ	(5.8)
12	YKLEIRTPQ	(5.6)
14	YRLKIRTPQ	(5.5)
8	YKLKIRTPR	(7.1)
31	YKLKIQTPQ	(4.0)
22	YKLKTRTPQ	(4.5)
20	YELKIRTPQ	(4.6)
7	YKLKIRAPQ	(7.9)
24	YKLKIRIPQ	(4.4)
15	HKLKIRTPQ	(5.5)
50	NKLKIRTPQ	(2.8)
9	YKPKIRTPQ	(6.6)
41	YKLKIRTPL	(3.2)
13	CKLKIRTPQ	(5.6)
33	YKLKIRTLQ	(3.7)
35	YKLKIRTSQ	(3.6)
Consensus	YKLKIRTPQ	

Rank	Sequence	Z score
2	LELLKASRW	(108.7)
19	LELLRASRW	(4.6)
16	LELLEASRW	(5.0)
46	LELLKVSRW	(3.0)
38	LELLKAPRW	(3.6)
45	LELLKALRW	(3.0)
17	LESLKASRW	(4.9)
21	LELPKASRW	(4.5)
23	LGLLKASRW	(4.4)
36	PELLKASRW	(3.6)
18	LELLKASRR	(4.8)
4	LQLLALSRT	(70.9)
25	LRLLALSRT	(4.4)
39	LQLLALSRA	(3.6)
47	LQLLAPSRT	(3.0)
32	PQLLALSRT	(3.9)
29	LQLPALSRT	(4.1)
3	LELVRRSPV	(89.0)
30	LELARRSPV	(4.1)
34	LELVRRSPA	(3.7)
26	LELVHRSPV	(4.1)
44	LELVRHSPV	(3.0)
42	LELVRCSPV	(3.2)
28	LEPVRRSPV	(4.1)
43	LELVRRSSV	(3.0)
49	LELVCRSPV	(2.8)
6	LALIKRQPL	(18.1)
5	LALLMRQRP	(19.0)
48	LAMLTRGRP	(3.0)
Consensus	LeLl..sr.	

Figure S1. Multiple sequence alignment from the top 50 peptide sequences with associated Z scores shown in brackets after three rounds of panning. Two peptide motifs isolated against USP11_DU were identified (the rank number is shown for each peptide). Left: Motif 1 peptide sequences; Right: Motif 2 sequences. The sequence alignment was generated using MultAlin (1). High consensus level residues (≥80%) are depicted in red, low consensus level residues (≥50%) are depicted in blue. Residues of ≤ 50% consensus level are depicted in black. All the positions in each sequence that are identical with the consensus are in upper-case, the other positions are displayed in lower-case.

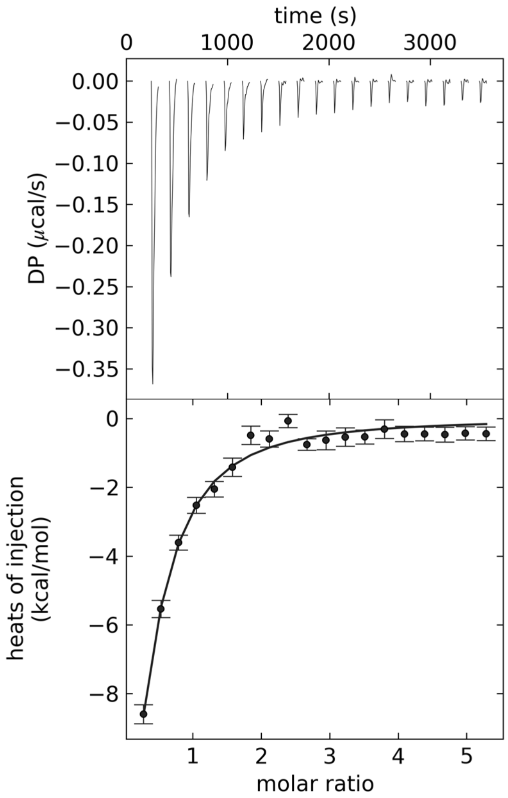

Figure S2. Full-length USP11 - FYLIR peptide interaction. Top panel: ITC data (power versus time curve) of FYLIR peptide (AEGEFYKLKIRTPR) titrated into full-length USP11 (FL-USP11); Bottom panel: integrated peak intensities plotted against the molar ratio. The dissociation constant of about 12.5 µM (n=1) for the full-length USP11–peptide interaction corresponds well to the one obtained for USP11_DU (Fig. 1D) after data fitting to a one site binding model disclosing that the binding site is available in FL-USP11.

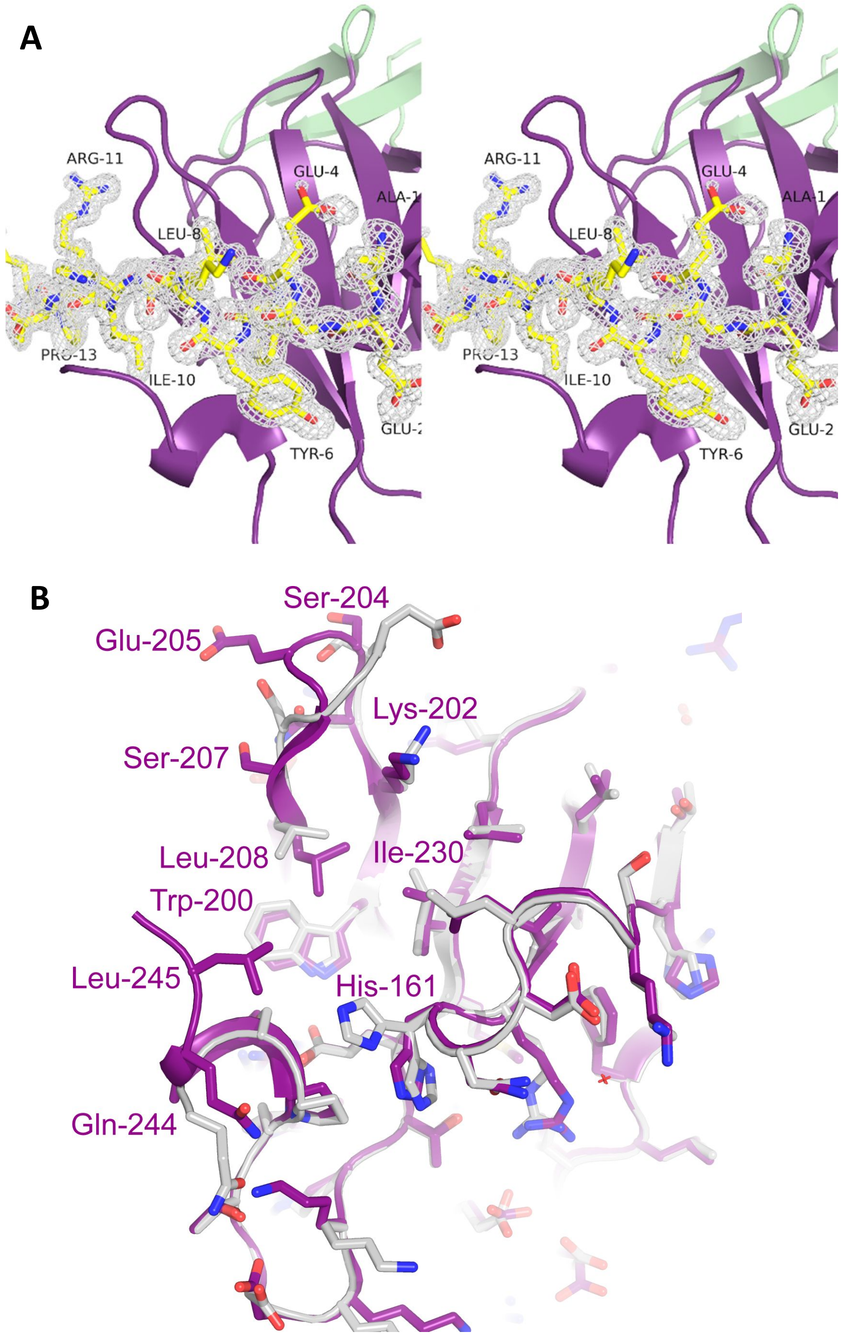

Figure S3. Electron density for FYLIR peptide and conformational changes in USP11 upon peptide binding. (A) Stereo-image of a mFobs-DFcalc omit electron density map calculated with the peptide removed contoured at 2.2 σ with the FYLIR peptide density shown in grey and corresponding final model in yellow stick representation. The USP11 UBL domain is depicted in purple and the DUSP domain in green. (B) Superposition of the crystal structures of the USP11 UBL domain in unliganded form (grey, PDB code 4MEL) and when in complex with the FYLIR peptide (purple; for clarity the peptide is not displayed). Residues that undergo conformational changes upon peptide binding are labelled.

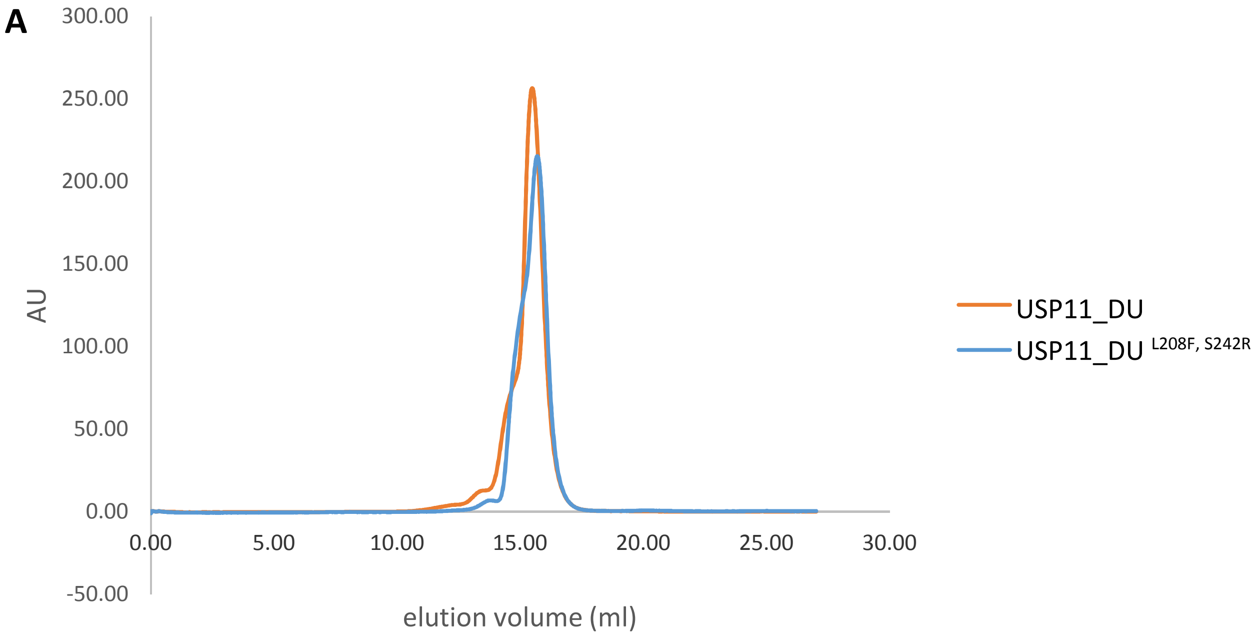

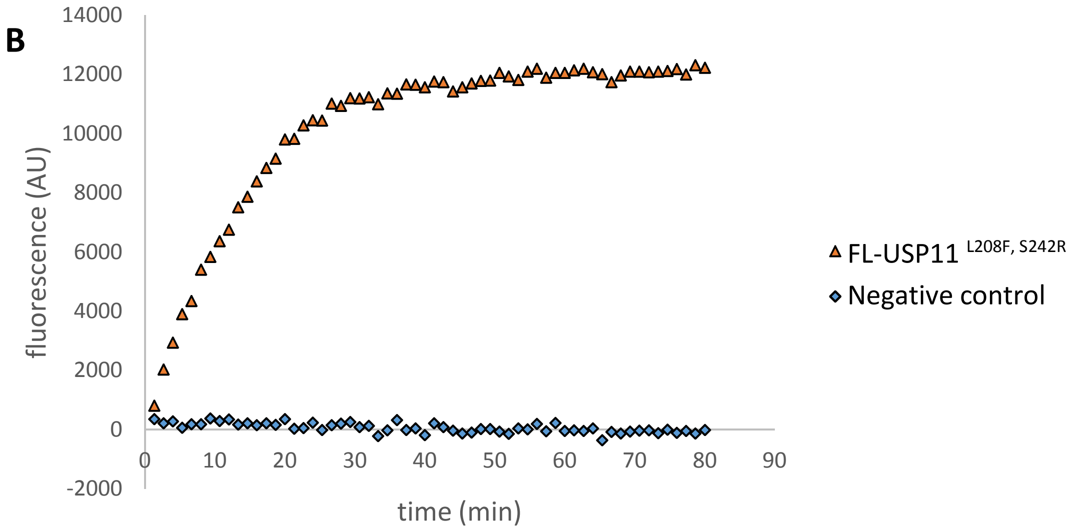

Figure S4. Characterization of USP11 $^{L208F, S242R}$. (A) Overlay of gel filtration traces of USP11_DU and USP11_DU $^{L208F, S242R}$ using a Superdex 200 10/300 GL column showing similar elution volumes of ~15.5 and ~15.7 ml in 50 mM Tris-Cl pH 7.5, 300 mM NaCl, 1% glycerol, respectively. This demonstrates that the mutant is folded and conformational changes are likely to be local to the mutation site, therefore not resulting in significant differences in the hydrodynamic radius. (B) Progress curve of hydrolysis of model substrate ubiquitin-AMC (750 nM) catalyzed by FL-USP11 $^{L208F, S242R}$ (110 nM; orange triangle) versus a negative control (no enzyme; blue diamond) in 50 mM Tris-Cl pH 7.5, 300 mM NaCl, 1 mM DTT reaction buffer demonstrating that the mutant is active (n=2 biological replicates) .

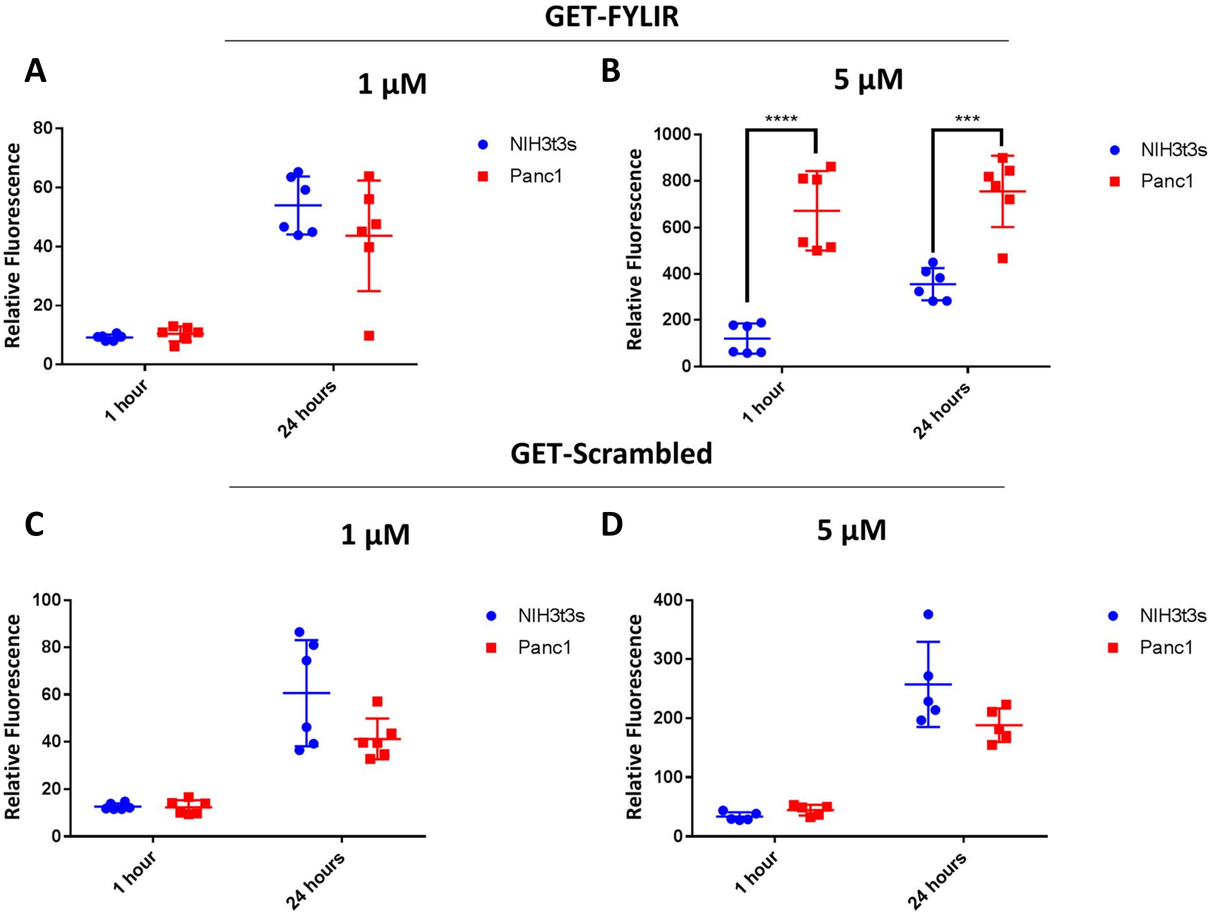

Figure S5. Effect of cell-type, duration and concentration of GET-FYLIR intracellular levels. The GET-FYLIR peptide that is TAMRA-tagged was delivered to cells at 1 μM (A) and 5 μM (B) for 1 or 24 hours. A control peptide (GET-Scrambled) was delivered to the cells at 1 μM (C) and 5 μM (D) concentrations for 1 or 24 hours. The intracellular concentration was assessed by flow cytometry. At a low concentration of GET-FYLIR (1 μM) similar intracellular concentrations regardless of the exposure time are observed in Panc1 and NIH3t3 cells. With a higher dose of GET-FYLIR (5 μM) there is a cell-type specific increase in the fluorescence levels within Panc1 cells which is not observed in NIH3t3 cells ($p<0.001$, $n=6$ biological repeats). Uptake of the scrambled control is similar in both cell lines independent of the dose or the exposure time.

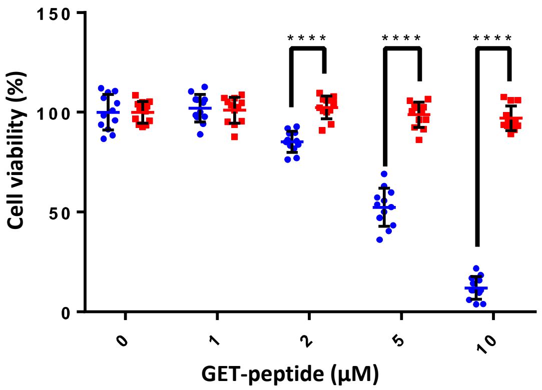

Figure S6. Effect of the FYLIR peptide on cell viability of Panc1 cells. Cell viability (Presto Blue assay) was assessed after 24 h incubation with GET-FYLIR (blue symbols) or GET-Scrambled (red symbols) at 0, 1, 2, 5 and 10 µM in Panc1 cells. Cell viability was expressed as percentage of cell viability ± s.d. (****$p<0.0001$, n=4 biological repeats, and 3 instrumental repeats per biological repeat were carried out, Sidak's multiple comparisons test).

Supporting references

1. Corpet, F. (1988) Multiple sequence alignment with hierarchical clustering. *Nucleic. Acids Res.* **16**, 10881-10890
